# Systems change for the social determinants of health

**DOI:** 10.1186/s12889-015-1979-8

**Published:** 2015-07-14

**Authors:** Gemma Carey, Brad Crammond

**Affiliations:** Regulatory Institutions Network, College of Asia and the Pacific, The Australian National University, Canberra, ACT Australia; Department of Epidemiology & Preventive Medicine, Monash University, Melbourne, Australia

## Abstract

**Background:**

Inequalities in the distribution of the social determinants of health are now a widely recognised problem, seen as requiring immediate and significant action (CSDH. Closing the Gap in a Generation. Geneva: WHO; 2008; Marmot M. Fair Society, Healthy Lives: The Marmot Review. Strategic Review of Health Inequalitites inEngland Post-2010. London; 2010). Despite recommendations for action on the social determinants of health dating back to the 1980s, inequalities in many countries continue to grow. In this paper we provide an analysis of recommendations from major social determinants of health reports using the concept of ‘system leverage points’. Increasingly, powerful and effective action on the social determinants of health is conceptualised as that which targets government action on the non-health issues which drive health outcomes.

**Methods:**

Recommendations for action from 6 major national reports on the social determinants of health were sourced. Recommendations from each report were coded against two frameworks: Johnston et al’s recently developed Intervention Level Framework (ILF) and Meadow’s seminal ‘12 places to intervene in a system’ (Johnston LM, Matteson CL, Finegood DT. Systems Science and Obesity Policy: A Novel Framework forAnalyzing and Rethinking Population-Level Planning. American journal of public health. 2014;(0):e1-e9; Meadows D. Thinking in Systems. USA: Sustainability Institute; 1999) (*N* = 166).

**Results:**

Our analysis found several major changes over time to the types of recommendations being made, including a shift towards paradigmatic change and away from individual interventions. Results from Meadow’s framework revealed a number of potentially powerful system intervention points that are currently underutilised in public health thinking regarding action on the social determinants of health.

**Conclusion:**

When viewed through a systems lens, it is evident that the power of an intervention comes not from where it is targeted, but rather how it works to create change within the system. This means that efforts targeted at government policy can have only limited effectiveness if they are aimed at changing relatively weak leverage points. Our analysis raises further (and more nuanced) questions about what effective action on the social determinants of health looks like.

## Background

Inequalities in the distribution of the social determinants of health are now a widely recognised problem, seen as requiring immediate and significant action [1, 2]. Since the Black Report’s release in 1980, representatives of public health have been making recommendations regarding how to best address inequalities in the social determinants of health [5]. These recommendations are uniformly directed to government, asking it to intervene in key areas – such as housing and education – and at key points in the life course, particularly early childhood [1, 2, 5].

Despite numerous national and international reports urging action, inequalities in the social determinants of health continue to grow in many countries [6, 7]. As a consequence, social determinants of health researchers have begun turning their attention to systems science to supply new insights into how to reduce the social inequalities that lead to health inequalities [8, 9]; “In order to understand drivers of population health outcomes and disparities, it is essential to learn about and understand the underlying systemic complexities that generate the outcomes we observe.” [10]. Similarly, McGibbon & McPherson argue that “Local, regional, national, and international systems of inequity are inextricably linked and cannot be ameliorated without an analytic focus on how these complex systems act together in a complex web of larger systems that coalesce to produce growing health and social inequalities” [11].

Taking a systems approach encourages a rethinking of organisations and system issues, including how actors behave in relation to them and are involved in their diagnosis and treatment [12–15]. Here, the emphasis is placed on understanding the ‘whole’ system, rather than focusing exclusively on individual components [12–14, 16–18].

In healthcare, a systems approach has been applied in a range of areas, including general practice [19], health service organisations [20] and health care systems [14]. A systems lens has also been used to tackle complex public health problems, such as tobacco control [21] and obesity [3, 15]. Such an approach enables us to examine system components and the intricate relationships between them, as well as elucidating the complexity of whole systems. The World Health Organisation (WHO) has stated that ‘systems thinking’ provides a more complete understanding of real-world settings and how to produce change [13]. The insights generated by this growing body of work is proving critical for the design and evaluation of interventions aimed at improving health equity [13].

In this paper we provide an analysis of recommendations from major social determinants of health reports emerging from the UK and WHO, using the concept of ‘system leverage points’. Previous analyses of recommendations for action on social and health inequalities in the UK have argued that recommendations in the most recent reports have changed very little from those in early reports, such as the Black Report [5, 22]. Similarities include strong emphases on public education, working conditions and the early years of life. [22]. By analysing recommendations from a systems perspective, we aim to unpack these findings by exploring the deeper nature of recommendations being made – drawing attention to *how* action is conceptualised, rather than what areas or levels it is aimed at (i.e., which parts of the life course, or government policy and action). From this analysis, we highlight ways in which recommendations could be more nuanced and effective for creating change in the complex systems that govern health outcomes.

## Methods

Major reports on the social determinants of health were sourced. These included the Black Report, Acheson Report, Commission on the Social Determinants of Health Final Report, the Strategic Review of Health Inequalities in the UK, the WHO Review of the Social Determinants of Health, and the EU Report on Health Inequalities [2, 5, 23–25]. Upon closer analysis, the Commission on the Social Determinants of health Final Report was excluded due to its broad, transnational focus. This focus incorporates such a diverse range of contexts that a recommendation, for example, to provide quality childhood education could require minimal intervention in one country but paradigmatic change in another. The recommendations therefore lacked the specificity required to be analysed from our systems perspective.

The recommendations from each report were coded against two frameworks: Johnston’s et al. recently developed Intervention Level Framework (ILF) and Meadow’s seminal ‘12 places to intervene in a system’ [3, 4]. Johnson et al’s ILF outlines five levels of systems change (see Table. 1), and was developed from Meadow’s more extensive framework (i.e., Meadows 12 categories have been collapsed into 5. Table 3 shows how the two frameworks map onto one another). Consistent with Johnston’s approach, recommendations which sought to address social, structural, environmental and other ‘upstream’ determinants were coded. Those focused solely on the actions of individuals were not. Recommendations were then coded to Meadow’s 12 leverage points, to enable deeper analysis. See Table 2 for a detailed description of Meadow’s leverage points. One minor adjustment was made to Meadow’s leverage points, which was to separate out changes to social structures and changes to build environment structures. This was done because of the fundamentally different nature between these interventions (and the differences in ease between the two, i.e., changing a social structure is less resource intensive than changing a physical structure such as city planning).

**Table 1:** Intervention level framework

Level	Description	Effectiveness
Paradigm	System’s deepest held beliefs	Difficult to intervene at this level but highly effective
	Source of system’s goals, rules and structures	
Goals	Targets that conform to the system’s paradigm and need to be achieved for paradigm to shift	Action at this level can change the aim of the system
System structure	Interconnections between system elements and subsystems	Action at this level will shift the system structure by changing system linkages and dynamics
Feedback and delays	Allows the system to regulate itself by providing information about the outcome of different actions back to the source of the actions	Actions at this level can create new feedback or increase gain around existing loops
Structural elements	Changes to physical elements of the system, its actors or subsystems	Easiest level at which to intervene.
		Many actions at the level are required to create system-wide change

**Table 2:** Places to intervene in a system (adapted from [25]

	Intervention point	Description
Information & control parts of system	1. Transcending paradigms	To keep oneself unattached in the arena of paradigms, and stay flexible, in order to see that no paradigm is ‘true’ (i.e., to know that paradigms exist).
	2. Paradigms	The mindset of a system refers to the deepest held beliefs of its members. From them, come shared social agreements about system goals, information flows, feedbacks, stocks, flows and other system components. Societies resist challenges to paradigms harder than any other types of change.
	3. Goals	The goals of the system can direct the behaviour of all the above system components. The goals of a system can be deduced by what it *does*. Often, people within systems do not recognise the overarching system goal.
	4. Self-organization	The power to add, change or evolve system structure. Systems change themselves (i.e., they are self-organising). The ability to self-organise is the strongest form of system resilience, as a system that can evolve can survive almost any change.
	5. Rules	The incentives, punishments or constrains in operation within the system. The rules of a system define its scope, boundaries and degrees of freedom.
	6. Information flows	The structure of who does and does not have access to information. Changing the structure of how information flows in a system means creating a new feedback loop, delivering new information to a place where it wasn’t going before and therefore changing behaviour as a result.
		A missing feedback loop is the most common cause of system malfunction.
	7. Reinforcing feedback loops	The strength of the gain of driving loops (i.e., virtuous or vicious cycles)
	8. Balancing feedback loops	The strength of the feedbacks relative to the impacts they are trying to correct. A complex system usually has numerous negative feedback loops, so it can self-correct under different conditions and impacts.
Physical structure of systems	9. Delays	The lengths of time relative to the rates of system change.
		A system cannot response to short-term changes if it has long-term delays. Delays are relative to the rates of change in the system state that the feedback loop is trying to control.
	10. Stock-and-flow structures	Physical system systems and their notes of intersection
	10.a Social systems	Networks of actors
	10.b Phsyical system	Build environment
	11. Buffers	The sizes of stabilizing stocks relative to their flows
	12. Numbers	Constants, parameters such as subsidies, taxes and standards

A framework synthesis approach was taken, whereby qualitative data in synthesised through a highly structured approach in order to provide numerically based charts [26]. Here, data were summarised on the basis of categories of intervention identified inductively from the data. In terms of coding, we adapted the early-stage methods of framework synthesis to summaries and then identify the type of recommendations that make up the various levels of system functions/intervention points [3]. That is, homogenous content was identified and categorized against each leverage point (firstly the ILF five leverage points, followed by Meadow’s 12 Leverage points). Consistent with Johnson et al’s original work in this field, our aim was to understand the broad types of interventions being advocated for/recommended within SDoH and how these ‘function’ within the system. Table 3 provides examples across intervention points and areas. In Table 4, we take one area of intervention (education) across all reports to demonstrate how recommendations were coded.

**Table 3:** Example recommendations and coding

Leverage point		Marmot review [2]	WHO review [23]	EU Marmot review [24]	Acheson report [22]	Black report [21]	Implications
ILF [20]	Meadows						
Paradigm	1,2	Develop and implement standards for a minimum income for healthy living	Improve the level and distribution of social protection according to needs to improve health and address health inequities.	None stated	None stated	None stated	Explicitly stated or inferred (i.e., paradigm change would be required for recommended change to occur)
Goals	3	Extending the role of schools in supporting families and communities and taking a ‘whole child’ approach to education	Provide universal high-quality and affordable early years, education and child care system.	Ensure actions to reduce health inequalities are included in the mainstream of all policies.	High priority is given to policies aimed at improving health and reducing health inequalities in women of childbearing age, expectant mothers and young children.	National health goals should be established and stated by government after wide consultation and debate. Measures that might encourage the desirable changes in people’s diet, exercise and smoking and drinking behaviour should be agreed among relevant agencies.	Changes to the goals of the system to make them more equitable
					Providing equitable access to effective care in relation to need should be a governing principle of all policies in the NHS		
System structure	4,5,6	Increase the proportion of overall expenditure allocated to the early years and ensure expenditure on early years development is focused progressively across the social gradient	Undertake regular reporting and public scrutiny of inequities in health and its social determinants at all governance levels, including transnational, country and local.	Consider additional actions that engage with a wider variety of sectors, such as on public safety, energy, sustainable development, agriculture, tourism, consumer protection, justice, immigration and finance.	A review of data needs to improve the capacity to monitor inequalities in health and their determinants at a national and local level.	General Household Survey steps should be taken to develop a more comprehensive measure of income.	Recommendations here reflected ‘joined-up’ action across sectors through information sharing, greater monitoring and data collection.
Feedback & delays	7,8,9	Providing work-based learning for young people and those changing jobs/ careers, including apprenticeships	Take action to develop systems and processes within societies that are more sustainable, cohesive and inclusive, focusing particularly on groups most severely affected by exclusionary processes.	Explicitly link health inequality objectives to existing cross-cutting strategies	Establishing mechanisms to monitor inequalities in health and to evaluate the effectiveness of measures taken to reduce them.	Boost evaluation research and statistical and information units	Closely linked to system goals. Included evaluation efforts, scaling up of programs and reorientation of funding.
					We recommend assessing the impact of employment policies on health and inequalities in health		
		Providing easily accessible support and advice for 16–25 year olds on life skills, training and employment opportunities					
Structural elements	10a,10b,11,12	Review and implement systems of taxation, benefits, pensions and tax credits to provide a minimum income for healthy living standards and facilitate upwards pathways	Ensure concerted efforts are made to reduce inequities in the local determinants of health through co-creation and partnership with those affected, civil society and a range of civic partners	Foster ‘health-in-all-policy’ and ‘whole-of-government’	Further investment in high quality training for young and long-term unemployed people	Resources to be allocated should be based upon the future planned share for different services including a higher share for community health.	Physical changes to subsystems, including the introduction of programs (e.g., seeking to change social network structures or the build environment)
				Ensure that coordinated actions are taken, across policy domains and for all social groups, which improve health across the causal pathways that affect health.	The provision of additional resources for schools serving children from less well off groups to enhance their educational achievement.		
							System parameters, such as income taxation
						A non-means-tested scheme for free milk should now be introduced beginning with couples with their first infant child and infant children in large families.

**Table 4:** Coding examples of education-related interventions

Intervention level framework	Meadows’ 12 leverage points	Example recommendation	Source
Paradigms	The power to transcend paradigms	None	
	The mindset or paradigm out of which the system — its goals, structure, rules, delays, parameters — arises	Provide good quality early years education and childcare proportionately across the gradient. This provision should be:	Marmot Review
Goals	The goals of the system	An integrated policy for the provision of affordable, high quality day care and pre-school education with extra resources for disadvantaged communities.	Acheson Report
	The power to add, change, evolve, or self-organize system structure	None	
	The rules of the system (such as incentives, punishments, constraints)	A statutory obligation should be placed on local authorities to ensure adequate day-care in their area for children under 5 and a minimum number of places the number being raised after regular intervals should be laid down centrally.	Black Report
System Structure	The structure of information flows (who does and does not have access to information)	Ensure concerted efforts are made to reduce inequities in the local determinants of health through co-creation and partnership with those affected, civil society and a range of civic partners.	WHO Review
Feedbacks and Delays	The gain around driving positive feedback loops	Further development of high quality pre-school education so that it meets, in particular, the needs of disadvantaged families. We also recommend that the benefits of pre-school education to disadvantaged families are evaluated and, if necessary, additional resources are made available to support further development.	Acheson Report
	The strength of negative feedback loops, relative to the impacts they are trying to correct against	Increase the availability of non- vocational life-long learning across the life course	Marmot Review
	The lengths of delays, relative to the rate of system change	Ensure actions are large enough in scale, of sufficient intensity and long enough in duration in order to have impact on levels of health inequalities.	EU Marmot
Structural Elements	The structure of material stocks and flows (such as transport networks, population age structures)	Further develop 'health promoting schools', initially focused on, but not limited to, disadvantaged communities.	Acheson Report
		Enact policies which promote moderate intensity exercise including: further provision of cycling and walking routes to school, and other environmental modifications aimed at the safe separation of pedestrians and cyclists from motor vehicles; and safer opportunities for leisure.	Acheson Report
	The sizes of buffers and other stabilizing stocks, relative to their flows	Provide good quality early years education and childcare proportionately across the gradient. This provision should be combined with outreach to increase the take-up by children from disadvantaged families	Marmot Review
	Constants, parameters, numbers (such as subsidies, taxes, standards)	Provision of additional resources for schools serving children from less well off groups to enhance their educational achievement. The Revenue Support Grant formula and other funding mechanisms should be more strongly weighted to reflect need and socioeconomic disadvantage.	Acheson Report
Not systems interventions		Further measures to improve the nutrition provided at school, including: the promotion of school food policies; the development of budgeting and cooking skills; the preservation of free school meals entitlement; the provision of free school fruit; and the restriction of less healthy food.	Acheson Report

In total, 168 recommendations were coded, once uncodeable recommendations were removed (*N* = 4). Recommendations deemed uncodeable had insufficient detail upon which a reasonable assumption could be made about what function the intervention would play in the system. For example ‘we recommend policies which will promote the material well being of older people.’ could apply to a very wide range of interventions. Coding was carried out by both authors and differences were discussed until a consensus could be reached.

## Results

**Fig. 1:**
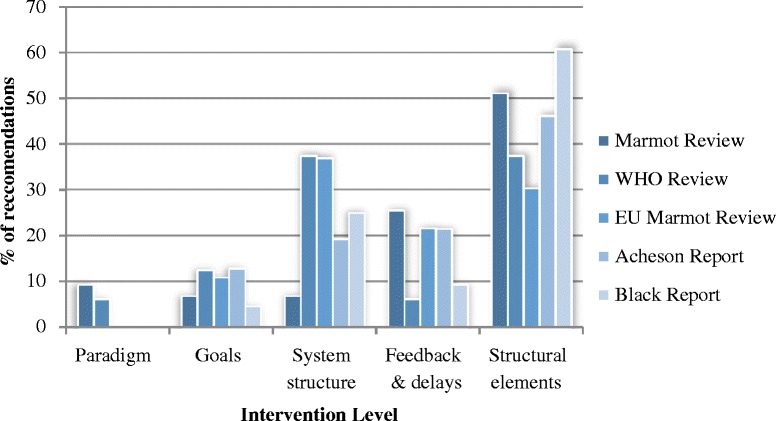
Distribution of recommendations using ILF

Figure 1 shows the distribution of recommendations by the five levels of intervention in Johnston et al’s ILF [3]. Recommendations were given more than one code, reflecting their multi-dimensional nature. Hence, values exceed 100 % in some instances. Recommendations coded at the level of structure (i.e., the physical structure of the system) were most common (*N* = 122). These included programs (which seek to change the structure of the system), changes to taxes and system ‘standards’ (see Table 3).

Increasing funding for particular interventions, whether new or scaling up existing interventions, and evaluation were coded as driving positive feedback loops (*N* = 24) (e.g. ‘Ensure progressive improvement in the availability and use of data needed to identify priorities, plan action, monitor trends and evaluate what actions are most effective’, would see monitoring and greater funding placed into effective interventions . Positive feedback loops can set up vicious or virtuous cycles – evaluation, increased funding for programs that are successful or attempts to ‘scale up’ interventions and programs are all efforts to maximise and increase the gains from driving existing positive feedback loops (see Table 3). For example, the UK Strategic Review of Health Inequalities includes the recommendation to ‘Increase the proportion of overall expenditure allocated to the early years and ensure expenditure on early years development is focused progressively across the social gradient’. By increasing spending on early years, opportunities are increased across the life-course, breaking cycles of disadvantage.

Recommendations that sought changes to system structure (*N* = 60) included efforts to change rules in order to create healthier environments (such as availability of healthy food ‘We recommend strengthening the CAP Surplus Food Scheme to improve the nutritional position of the less well off.’ [Acheson Report]), or system rules which drive health disparities (such as the rules which determine funding allocation to particular social determinants of health or life course-related issues). Changes to system structure also included the flow of information within the system, i.e., giving agencies access to data and reporting, but also linking them different in networks in order to provide new opportunities for insight and action (those requiring changes to social network structures were also coded as 10a (*N* = 42).

**Fig. 2:**
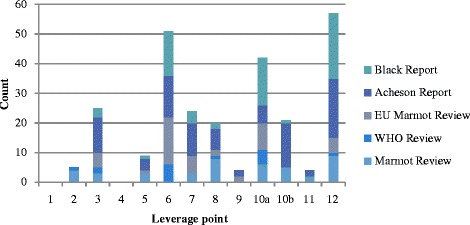
Frequency of recommendations against Meadow’s 12 leverage points

The results from the second level of coding are shown in Fig. 2. Here, results are displayed in the form of a count (i.e., number of times a given code appeared per report). Coding to the full 12 of Meadow’s Leverage Points (upon which the ILF is based) enabled a closer analysis of which intervention points were recommended or missed, and insight into changes over time. Within category 10 – structural changes to the system – we draw a distinction between changes to the built environment (10b) and changes to social network structures (10a). Recommended changes to social network structures were far more common than to the build environment.

The more detailed analysis presented in Fig. 2 reveals several shifts in the types of recommendations being made between early and later reports. For example, changes to system parameters (leverage point 12) – an easy, but relatively weak intervention point – have become less common. Moreover, early reports did not include recommendations aimed at shifting paradigms, but the later Marmot Reviews do (leverage point 2). This is despite the fact that changes to paradigms are significantly harder to achieve than lower lever intervention points.

## Discussion

In social determinants of health research, what is sometimes referred to as ‘upstream’ change – that is change within government and policy – is seen as a more powerful and effective intervention point for addressing the social determinants of health than ‘downstream’ measures which target communities or individuals [2, 27, 28]. This is particularly so when action centres on the ‘determinants of health’, rather than health itself [29, 30]. Upstream action is increasingly emphasised over and above programs or interventions aimed at individuals or community groups [27, 30, 31]. This is because the social determinants of health are now understood to be affected by the organisation of material and social resources amongst the members of societies, which is best addressed through government action [30].

Interestingly, the systems frameworks developed by Meadows [4] and Johnston et al. [3] do not map neatly onto the upstream-downstream dichotomy which now dominates much discussion on population health interventions for the social determinants of health. Rather than the level at which an intervention in made, systems frameworks draw attention to the *way* we intervene. That is, how we intervene in a system can be much more important than where we intervene; interventions made within government can still fail to take hold thereby generating few positive outcomes [29].

For example, recommendations that called for joined-up action between different policy actors and between different levels within service delivery systems (i.e., a linking of different parts of government, or government and other sectors – sometimes referred to as ‘whole of government approaches’ or ‘horizontal government’ [29]) were amongst the most common recommendations, particularly in later reports. The EU Report on Health Inequalities, for example, called for ‘all governmental levels to liaise and cooperate with other sectoral policies and invest smartly in specific health inequality measures’. This constitutes joined-up action in the sense that different parts of government need to connect with and work closely with other departments (see Table 3 for further examples). An exception to the popularity of recommendations for joined-up action is the UK Marmot Review into Health Inequalities, which did not include explicit recommendations for joined-up action. However, this reflects a limitation of our data extraction methods – the UK Marmot Review is premised on the notion that joined-up action is required to deliver on all recommendations contained in the review. This is reflected throughout the report and in the implementation and measurement plans.

While joined-up government/whole of government approaches are seen as a powerful intervention point, they sit towards the lower (less effective) end in Meadow’s scale. This type of action was coded as changes to actor network structures (10a) and information [6]. Physical system structures (built or otherwise) are tricky to change. In the case of actor networks, the adaptive nature of systems can come into play to mitigate outcomes that could be precipitated by such changes. That is, the self-organizing properties of systems means that they can quickly adapt to changes made at low intervention points, causing these changes to ‘wash out’ and have little effect. Recent research on the type of joined-up government/whole of government changes suggested by these recommendations indicates that more often than not these ‘upstream’ interventions do wash out and the system returns to the status quo [29]. The collection and reporting of information, implicit in joined-up efforts, can be an effective leverage point but only under particular circumstances.

Collection and reporting of information on its own is not enough to generate substantive change. To be effective, information flows must be restored to the right place in the system and in a compelling form [4]. Meadows uses the analogy of a pilot, who receives information on the state of the aircraft and is positioned to act swiftly on this information. Moreover, if a pilot does not act he/she will immediately feel the repercussions of this failure to act. Hence, the type of data collection systems that are recommended across the various reports coded (see Table 3) need to be integrated into the system in such a way as to force decision-makers to act. Viewing data collection and reporting through a systems lens can therefore make the difference between a relatively weak action in terms of systems change and a very powerful one.

Recommendations that addressed feedback loops were common (*N* = 44). Feedback loops can be balanced or reinforced. For example, if left unchecked the flu creates reinforcing feedback loops – the more people who catch the flu, the more they infect others. Balancing this feedback loop then would be the administration of flu shots. How effective this is depends on the strength of the balancing effort compared to the force it is trying to correct. If only a small number of individuals get flu shots, or if the shot itself has only a limited impact on whether individual catch the flu, the power of its balancing effect will be too small in comparison to the force it is countering and the flu will continue to spread. Here, a systems lens draws attention to the strength of the feedback mechanisms put in place, relative to the problem they are trying to address. Taking an example from the Marmot review, the recommendation to provide support and advice to young people regarding training and employment opportunities will only create pathways into good employment if there are (a) sufficient number of training placements and jobs are available and (b) other structural barriers are minimised. Otherwise, the corrective force of this intervention will be too weak to counter the broader issues which mean young people do not take up training opportunities (such as family or social problems, or a lack of training placements).

In coding to Meadow’s full twelve leverage points, we found several powerful but underutilised leverage points. Few recommendations argued for changes to rules in the system. Rules define the boundaries, or scope of the system. When dealing with inequalities in the social determinants of health, rules become critically important. A simple example of this is how much wealth we allow individuals to accumulate. If this is unlimited, disparities are free to widen. If we cap the amount of wealth any individual can posses, we stop growth at the top end of the social gradient. As Meadows contends, “If you want to understand the deepest malfunctions of systems, pay attention to the rules and to who has power over them” [4]. In our example, these rules are taxes that favour the wealthy. It is worth noting that, while powerful, these types of changes to system rules can be socially and politically difficult to achieve. This is likely to be particularly so in countries with countries that operate under state regimes that favour individualism and fewer government funded services and support (i.e., liberal versus social democratic regimes) [31–33].

No recommendations were coded as a the power to add to, change, or evolve system structure (leverage point 4 in Meadow’s framework). Systems are naturally self-organising, where complex behaviour emerges from relatively simple building blocks or rules (for example, DNA). Using this self-organising nature to one’s advantage can be a powerful leverage point. A focus on the self-organising tendency of systems can generate highly sophisticated and nuanced approaches to change.

Social determinants of health advocates frequently call for changes to policy. Yet, this is commonly done in broad terms, such as ‘We recommend policies to improve the quality of jobs, and reduce psychosocial work hazards’ (Acheson Report) or ‘Public policy—both national and global—should change to take into account the evidence on social determinants of health and interventions and policies that will address them’ [34]. However, understanding the self-organising properties of systems, and the role of feedback loops in enabling this self-organisation, means we begin to think more carefully about the type of policy changes we recommend. The literature on systems science and public policy argues for ‘adaptive’ or ‘learning’ policies. A dynamic, self-adjusting feedback system cannot be governed by a static, unbending policy. In fact, static policies often fail to produce their intended effect as the dynamic system shifts around them. The Australian government’s taxation increase on ready-to-drink spirits-based alcoholic beverages (referred to as alcopops) sought to decrease harmful drinking by, particularly, young women. The targeted increase saw consumption of other drinks rise [35] and no change in alcohol-related violence [36]. Most relevant is the observation by Doran and Digiusto [37] that ‘it is impossible to know how much of the [consumption] changes were due to the tax, to the ‘global financial crisis’, to adaptive marketing by the alcohol industry, to the Government’s national binge drinking strategy, to mass media coverage of these issues or to other factors.’

Learning, or adaptive, policies change depending on the state of the system [4, 38, 39] . For example, an adaptive education policy would make the proportion of government funding for private schools contingent upon the performance of public schools. When public schools perform well, private schools receive more funding. When they perform poorly, government funds for private schools decreases. This type of adaptive policy changes as the system changes, but also uses the self-organising principles of the system to achieve a particular outcome (i.e., more equality in school outcomes between public and private systems) [38]. Here, the rules and incentives are bent towards favourable action in terms of achieving the goal of reducing the inequalities which stem from tiered education systems. These built in policy adjustments can speed up the process of responding to emergent conditions within the system [39, 40].

Finally, it is worth noting the limitations of this study. The research only considered a subset of all SDOH reports – concentrating on the UK context in the main. Reports from other countries, such as Brazil and other parts of Latin America where action on the SDOH has occurred, could yield different results and would be a worthwhile area of future investigation. These different contexts may require, or potentially enable, different types of action to be taken. It is also worth noting that what is contained in the recommendations of the reports analysed is not necessarily representative of the aspirations of the field of SDOH research as a whole. The reports are produced within particular political contexts which constrain the types of recommendations that can be made. All of the Reports we analysed display a tendency towards centrist policies endorsing neither neoliberal, market-based solutions nor highly socialised market-opposed interventions. These constraints may have some effect on the content of the recommendations but they need not effect the types of leverage points targeted. There is no reason why the right or left of politics would be more likely to target the rules of the system or its goals. Were these constraints lessened, therefore, our analysis of ‘how’ we intervene upstream would remain relevant.

## Conclusion

Powerful and effective action on the social determinants of health is increasingly conceptualised as that which targets government action on non-health issues which drive health outcomes. When viewed through a systems lens, it is evident that the power of an intervention comes not from where it is targeted, but rather how it works to create change within the system. This means that efforts targeted at government policy can have limited effectiveness if they are aimed at changing only relatively weak leverage points (such as changes to network structures) (see, for example, [29].

Our analysis raises further questions about what effective action on the social determinants of health looks like. For example, should ‘upstream’ action seek high leverage points, such as the goals of the system? While difficult, these efforts could have a profound effect on social and health inequalities.
